# Mechanical Properties of the Composite Material consisting of β-TCP and Alginate-Di-Aldehyde-Gelatin Hydrogel and Its Degradation Behavior

**DOI:** 10.3390/ma14051303

**Published:** 2021-03-09

**Authors:** Michael Seidenstuecker, Thomas Schmeichel, Lucas Ritschl, Johannes Vinke, Pia Schilling, Hagen Schmal, Anke Bernstein

**Affiliations:** 1 G.E.R.N. Tissue Replacement, Regeneration & Neogenesis, Department of Orthopedics and Trauma Surgery, Medical Center-Albert-Ludwigs-University of Freiburg, Faculty of Medicine, Albert-Ludwigs-University of Freiburg, Hugstetter Straße 55, 79106 Freiburg, Germany; thomas033@web.de (T.S.); lucas.ritschl@uniklinik-freiburg.de (L.R.); pia.schilling@uniklinik-freiburg.de (P.S.); hagen.schmal@uniklinik-freiburg.de (H.S.); anke.bernstein@uniklinik-freiburg.de (A.B.); 2 Institute for Applied Biomechanics, Offenburg University, Badstraße 24, 77652 Offenburg, Germany; vinke@hs-offenburg.de

**Keywords:** mechanical properties, degradation behavior, β-TCP, ADA-gelatin gels, fracture strength

## Abstract

This work aimed to determine the influence of two hydrogels (alginate, alginate-di-aldehyde (ADA)/gelatin) on the mechanical strength of microporous ceramics, which have been loaded with these hydrogels. For this purpose, the compressive strength was determined using a Zwick Z005 universal testing machine. In addition, the degradation behavior according to ISO EN 10993-14 in TRIS buffer pH 5.0 and pH 7.4 over 60 days was determined, and its effects on the compressive strength were investigated. The loading was carried out by means of a flow-chamber. The weight of the samples (manufacturer: Robert Mathys Foundation (RMS) and Curasan) in TRIS solutions pH 5 and pH 7 increased within 4 h (mean 48 ± 32 mg) and then remained constant over the experimental period of 60 days. The determination surface roughness showed a decrease in the value for the ceramics incubated in TRIS compared to the untreated ceramics. In addition, an increase in protein concentration in solution was determined for ADA gelatin-loaded ceramics. The macroporous Curasan ceramic exhibited a maximum failure load of 29 ± 9.0 N, whereas the value for the microporous RMS ceramic was 931 ± 223 N. Filling the RMS ceramic with ADA gelatin increased the maximum failure load to 1114 ± 300 N. The Curasan ceramics were too fragile for loading. The maximum failure load decreased for the RMS ceramics to 686.55 ± 170 N by incubation in TRIS pH 7.4 and 651 ± 287 N at pH 5.0.

## 1. Introduction

According to the Federal Statistical Office of Germany, demographic change in Germany is becoming increasingly acute. Every second person in Germany today is over the age of 45, and every fifth person, over the age of 66 [1]. The same applies to the European Union. Here, too, one in five people is older than 65 [2]. These changes not only have consequences in our society and environment but also in the field of medicine. In 2019, 194,453 artificial hip joint implants were performed in German hospitals [3]. According to a Eurostat study for 2019, Switzerland, Austria, Belgium, and Finland are at the same high level with 275 to 300 hip replacements per 100,000 inhabitants as Germany with 311 [4]. When artificial hip joints have been implanted in the human body, the device is expected to have an average life of at least 15 years. However, the service life of an artificial knee joint is only 10 to 12 years [5]. Due to the aging of the implants, as well as abrasion of the plastic inlay, revision operations are often necessary. The reasons for revision surgery include complications, such as infections [5]. *Staphylococcus aureus* is currently still considered the main cause of implant-associated infections as well as acute and chronic osteomyelitis [6]. Therefore, the highest priority should be to stop the infection and thus the spread of the bacteria to initiate optimal wound healing [7,8,9]. One possibility for treating the infection is local antibiotics. Local application offers the advantage that high local antibiotic levels can be achieved at the site of the event while avoiding the harmful effects of the antibiotics used on the rest of the organism [10]. Carrier systems for the local application can be divided into two groups: degradable and non-degradable systems [11]. Carrier systems should promote an initial rapid local release of antibiotics and growth factors and promote the healing process. Calcium phosphate (CaP) ceramics have established themselves as an exceptionally good implant material, as they do not cause toxic or foreign body reactions [12]. Human bone consists mainly of inorganic materials, such as calcium compounds, especially hydroxyapatite (HA), and organic substances, such as collagen [13,14,15]. Due to the chemical similarities of the materials to the mineral phase of human bone, calcium compounds are frequently used for biomedical applications. In one of our previous studies, we were able to show that alginates are very well suited for delaying drug release [16]. However, since alginates can only be degraded by the human body to a limited extent, an alternative was required. In their work, Sarker et al. [17] described the modification of alginate so that it can be crosslinked with gelatin. Furthermore, the release experiments presented using alginate-di-aldehyde (ADA) layers were promising. This work aimed to investigate the influence of ADA-gelatin hydrogels in a microporous CaP ceramic, which is intended to serve as a drug carrier (for sustained drug release) for the treatment of bone infections, and determine the mechanical strength and the degradation behavior of the composite. 

## 2. Materials and Methods

### 2.1. Materials

Gelatin 300 Bloom (Art. No. G2625) was kindly provided by Gelita AG (Eberbach, Germany). Alginic acid for microbiological applications (Art. No. 71238), sodium periodate (Art. No. S1878), alginate (Art. No. A2033), potassium chloride (Art. No. P5405), magnesium chloride anhydrous (Art. No. M8266), calcium chloride dihydrate (Art. No. C7902), sodium sulfate (Art. No. 746363), ethylene glycol (Art. No. 324558) and TRIS (Art. No. T6066) were purchased from Sigma–Aldrich (Merck, Switzerland). Sodium chloride (Art. No. 3957.3), sodium hydrogen carbonate (Art. No. 6885.2), disodium hydrogen phosphate dihydrate (Art. No. T876.1), and 1 M hydrochloric acid (Art. No. K025.1) were purchased from Carl Roth (Karlsruhe, Germany). 

### 2.2. β-TCP Ceramics

The β-TCP ceramics used in this work were produced according to our specifications by the Robert Mathys Foundation (RMS). Eighty grams of α tricalcium phosphate (α- TCP; Ca_3_(PO_4_)_2_) and 20 g tricalcium phosphate (Art. No. 102143, Merck, Switzerland) were mixed with a 60.0 ± 0.2 g solution of 0.2 M Na_2_HPO_4_ and 1% polyacrylic acid (Art. No. 81132, Fluka, Switzerland; Mw = 5.1 kDa). The paste was poured into a plastic syringe after 2.5 min of intensive stirring. The plastic syringe had a diameter of 23 mm and a length of 70 mm. After 45 minutes, the paste was covered with 10 mL of phosphate-buffered saline (PBS) (Item No. P5368, Sigma, USA), pH 7.4 solution, and incubated for 3 days at a temperature of 60 °C. The green bodies were then dried at the same temperature and sintered for 4 h at 1250 °C with a heating and cooling rate of 1°C/min. Afterward, the ceramic cylindrical-shaped bodies were cut to a diameter of 7 mm and a length of 25 mm. As the last step, the ceramic plugs were washed in an ethanol bath and calcined at 900 °C to remove all wear particles and organic residues by combustion [18]. In addition, for comparison purposes, commercially available β-TCP ceramics, cylindrical Cerasorb M moldings with a diameter of 7 mm and a length of 25 mm, were purchased from Curasan (Kleinostheim, Germany).

### 2.3. Characterization of the β-TCP Ceramics

#### 2.3.1. Weight and Dimensions

The β-TCP ceramics (RMS and Curasan) were weighed and measured. The sample dimensions were measured with a Burgwächter PS 7215 digital caliper gauge (Burg-Wächter, Wetter-Volmarstein, Germany). The scaffolds were weighed with a Sartorius Practum analytical balance (Sartorius, Goettingen, Germany). Each sample was measured at least 3 times.

#### 2.3.2. Microstructure and Elemental Analysis

The microstructure of the β-TCP ceramics (RMS and Curasan) was performed by means of an ESEM (FEI Quanta 250 FEG) (FEI, Hilsboro, OR, USA) analysis. The ceramic dowels which had been previously in the buffer solutions were first dried completely at 200 °C in a UFP-500 Memmert drying oven (Memmert, Schwabach, Germany) for 4 h. Afterward, the samples were glued to the sample holders with a double-sided carbon conductive pad (Plano, Wetzlar, Germany) and analyzed by environmental scanning electron microscopy (ESEM). An acceleration voltage of 10 kV was used for the ESEM analysis. The composition of the sintered ceramics was defined via energy-dispersive X-ray spectroscopy (EDX) (Philips ESEM XL 30 FEG) (Philips, Amsterdam, Netherlands) and by XRD (Bruker D8 Advance) (Billerica, MA, USA). For the EDX studies, the cylindrical samples were broken in the middle with cutting pliers, and the fracture surface was analyzed. The measurement conditions were 12 kV accelerating voltage and 100 s measurement time (live time). For XRD analysis on the Bruker D8 Advance, the samples were ground with an agate mortar. Measurement conditions were Bragg–Brentano geometry, equipped with Cu anode and secondary graphite monochromator, scintillation counter, 40 kV/40 mA, 1°2theta/min, step size 0.02°2theta.

For micro-computed tomography (µCT) measurements, a µCT 50 from SCANCO (SCANCO Medical AG, Bruettisellen, Switzerland) with a detector having a 2 µm resolution was used. The samples were cut to a thickness of 3 mm. To remove the abrasion from the processing, the samples were then cleaned three times (ethanol/water/water) in an ultrasonic bath for 15 min each. The set parameters during the measurement were 90 kV; 4 W, 44 µA at a resolution of 2 µm, an integration time of 5000 ms. One thousand, five hundred sectional images of 180° each were recorded. The µCT measurements were performed by SCANCO Medical AG in Bruettisellen/Switzerland.

#### 2.3.3. Porosity

The porosity of both β-TCP ceramics (RMS, Curasan) was measured with a Porotec 140/440 porosimeter (Porotec GmbH, Hofheim, Germany). To make sure that there was no water left in the specimens, they were annealed for 24 h at 105 °C. For the determination of pore sizes with a diameter of 1000 µm–1.4 µm, a Pascal 140 low-pressure porosimeter was used (pressure was built-up up to 0.1 KPa). Afterward, the specimens were transferred into a Pascal 440 high-pressure porosimeter (pressure was built-up up to 400 MPa) for the measurement of the pore sizes from 1.4 µm to 1.8 nm.

#### 2.3.4. Surface Roughness

To determine the surface roughness, the samples were examined using a KEYENCE 3D Laser Scanning Microscope VKX-210 (KEYENCE, Osaka, Japan). The surface roughness (S_a_) was determined using KEYENCE VK Analysis software (KEYENCE, Osaka, Japan) Version 3.5.0.0. A total of 5 different samples were analyzed: Curasan, RMS, RMS in simulated body fluid (SBF), RMS in TRIS pH 7.4, and RMS in TRIS pH 5.0 ceramic. Several images were taken and analyzed from each sample. The specimens were examined in a 1000× magnification.

### 2.4. Preparation of the Hydrogels

The aim was to investigate not only the influence of ADA-gelatin gels on the fracture strength of composites but also the influence of alginate gels. The latter served as a control besides the empty ceramics. Before hydrogel production, all starting materials and the individual parts of the loading chamber were sterilized with an autoclave (Varioclav^®^, HP-Medizintechnik GmbH, Munich, Germany). The stirring plate, the magnetic stirrers, as well as the vacuum-proof tubes, including the surfaces inside the sterile bench, were sterilized with 70% Ethanol.

#### 2.4.1. Alginate

Alginate sol with 1.5% *w*/*v* alginate (Art. No. A2033) was prepared. Before use, sterile filtration was performed using syringe filters (0.20 µm).

#### 2.4.2. Alginate-Di-Aldehyde (ADA)

ADA was produced according to the method developed by Sarker et al. [17]. For this purpose, 5 g of sodium alginate (Art. No. 71238) was dissolved in 25 mL ethanol (99.8%), and 1.605 g sodium periodate was dissolved in 25 mL double distilled water. The sodium periodate solution was then added dropwise to the alginate–ethanol suspension under light exclusion with constant stirring (250 RPM). For the oxidation reaction, stirring was carried out for 6 h under the exclusion of light (i.e., the beaker was wrapped with aluminum foil). The reaction was stopped with 5 ml ethylene glycol, and then stirring was continued for 30 min. The ADA was dialyzed for 7 days against double distilled water to remove any remaining sodium periodate using the dialysis system Spectra/Por (Repligen, Boston, MA, USA) with standard RC dialysis membranes (6–8 kD MWCO). The water was changed twice a day. After dialysis, the ADA was dried in a lyophilization FreeZone Plus (Labconco, Kansas City, MO, USA) for another seven days. A 5% *w*/*v* ADA-solution was prepared by dissolving ADA in double-distilled water and stirred at 250 RPM overnight.

#### 2.4.3. Gelatin

A gelatin solution with 5% *w*/*v* was prepared. The beaker containing the solution was sealed with parafilm and incubated at 40 °C in a UFP-500 drying oven (Memmert, Schwabach, Germany). Before use, sterile filtration was performed using syringe filters (0.20 µm).

#### 2.4.4. ADA-Gelatin

The ADA-gelatin gel was prepared directly before the loading process by mixing 1:1 ADA gel with gelatin solution and stirring at 250 RPM for 1 min.

### 2.5. Preparation of the Buffers

#### 2.5.1. Simulated Body Fluid (SBF)

Simulated body fluid (SBF) (1000 mL) was prepared as described by Jalota et al. [19]. The chemicals used to prepare the SBF and the quantities which were added are outlined in Table 1. A beaker of deionized water was placed on a magnetic stirrer at 37 °C. Each chemical was weighed using electronic scales and added to the deionized water in the order presented in Table 1. An electronic pH meter (Mettler Toledo, EL20, Columbus, OH, USA) was then used to measure the exact pH of the solution, and hydrochloric acid (HCl) was added slowly until the solution reached a pH of 7.4. The beaker was then covered with aluminum foil and left on the magnetic stirrer overnight. The following day, the solution was filtered through a 0.2 µm (pore size) filter and sealed under sterile conditions.

#### 2.5.2. TRIS–Buffer

According to ISO EN 10993-14, 26.5 g TRIS was dissolved in 1000 mL double distilled water. For further experiments, the pH values 7.4 and 5.0 were adjusted with 1 mol/L HCl.

### 2.6. Loading via Flow Chamber

The loading of the porous ceramics with the hydrogels was carried out, as we previously described [16,20], by means of a flow chamber developed by our group. By means of this flow-through chamber, it is possible to achieve a loading of porous ceramics in a flow which is achieved by a pressure difference between the reservoir tank (normal pressure) and the flow-through chamber (low vacuum of 50 mbar). The ceramics were embedded in a silicone seal and placed in the flow chamber. This is important because the gel should only be drawn in via the front surface of the ceramic. In addition, the silicone seal was intended to prevent the gel from taking the path of least resistance and escaping directly after the end face on the side of the ceramic. The loading of the ceramics was successful when gel leaked into the other side of the loading chamber.

### 2.7. Degradation Tests

#### 2.7.1. Degradation of β-TCP Ceramics

For the degradation tests, the samples were stored for 60 days according to the standard protocol in ISO EN 10993-14 in 5 mL TRIS buffer pH 7.4 each. To simulate the conditions in the human body caused by inflammation, the experiment was repeated with TRIS buffer pH 5.0. The samples were weighed before and after loading and every 2 days during the experiment by means of a precision balance, Kern PCB 250-3 (KERN & SOHN GmbH, Balingen, Germany). The incubation in SBF was 30 days, according to our previous studies [21,22]. All experiments (TRIS + SBF) were performed at 37 °C by using a Memmert drying oven UF500 (Memmert, Schwabach, Germany).

#### 2.7.2. Determination of the Protein Concentration Out of ADA-Gelatin Hydrogel

The degradation of the ADA-gelatin gel was determined using the Bradford Test, the ROTI® Nanoquant assay (Carl Roth, Karlsruhe, Germany). For this purpose, the TRIS buffer (pH 5 and pH 7) was completely exchanged weekly and replaced with new TRIS buffer. The collected samples were frozen at −20 °C. To determine protein concentration, a calibration curve was constructed using known bovine serum albumin (BSA) (Albumin Fraction V, Carl Roth, Karlsruhe, Germany) concentrations. The amount of protein was quantified using a UV-Vis spectrometer (Spectrostar nano, BMG LabTech, Ortenberg, Germany) at 590 nm and 450 nm. 

### 2.8. Compression Test

The compression test was performed using the Zwick Z005 universal testing machine. The testing machine was controlled, and data recorded using Zwick testXpert II (Version 3.7.1) (Zwick, Ulm, Germany). One hundred and eighty-five specimens were examined. The classification of the specimens is shown in Table 2.

The following parameters were set for the compression tests on the universal testing machine (see Table 3).

The compression test was completed when the maximum deformation, the force limit of 5000 N, or the maximum deformation of 50%, was reached. The maximum failure load and the compression strength should be determined.

### 2.9. Statistics

Data are expressed as mean ± standard deviation of the mean and were analyzed by one-way analysis of variance (ANOVA). The level of statistical significance was set at *p* < 0.05. For statistical calculations, the Origin 2020 Professional SR1 (OriginLab, Northampton, MA, USA) was used.

## 3. Results

### 3.1. Dimensions and Weight

The ceramic dowel was produced according to our specifications by the Robert Mathys Foundation (Bettlach, Switzerland). The dowel used for this project had the following dimensions: length 6.03 ± 0.12 mm diameter: 6.99 ± 0.01 mm and weight 0.368 ± 0.013 g (N = 185). The commercially available Curasan dowels had a length of 5.96 ± 0.04 mm, a diameter of 6.95 ± 0.02 mm, and a weight of 0.25 ± 0.08 g (N = 45).

### 3.2. Microstructure and Elementary Analysis

#### 3.2.1. Microstructure by Means of ESEM

**Figure 1:**
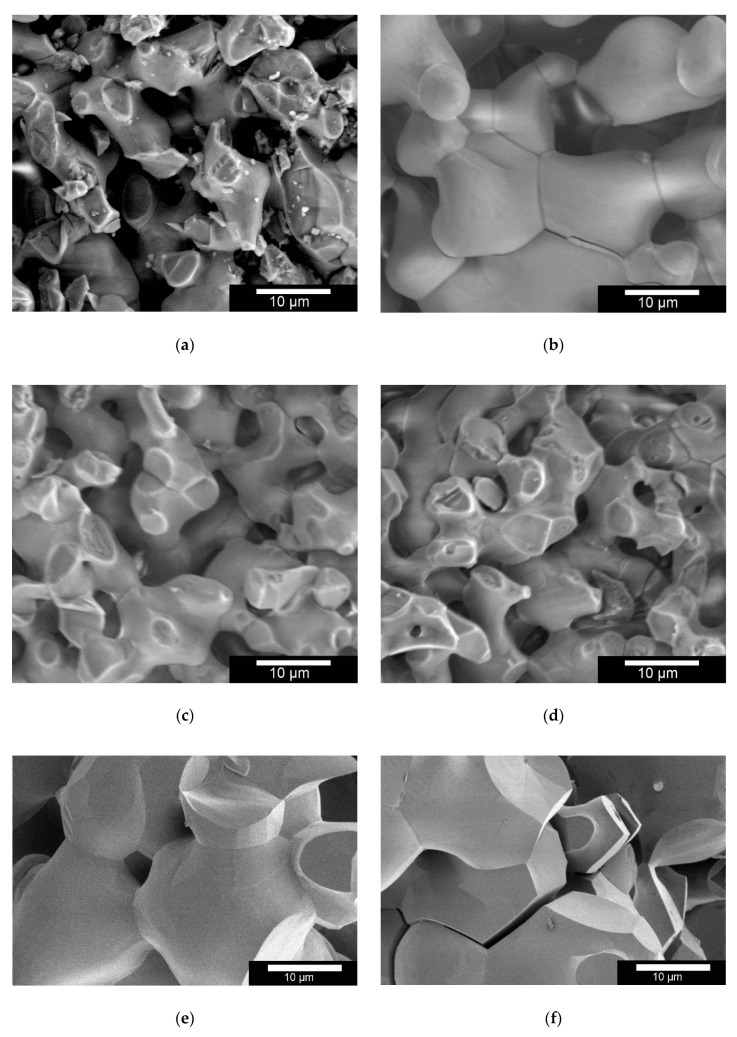
ESEM images of (**a**): Robert Mathys Foundation (RMS) and (**b**): Curasan β-TCP Ceramics; ESEM images of RMS Ceramics in TRIS buffer: (**c**): pH 5.0; (**d**): pH 7.4; Curasan Ceramics in TRIS buffer: (**e**): pH 7.4; (**f**): pH 5.0; Images were taken with an FEI Quanta 250 FEG and (**e**,**f**) FEI Scios, acceleration voltage 10 kV, HFW of 46.6 µm.

Compared to the ceramics produced for us by the RMS, the macroporous Curasan ceramics showed significant differences in microstructure (FIJI Version 1.53d). The pore sizes and strand widths of the two β-TCP ceramics exhibited a mean strand width of 7.1 ± 2.2 µm for the RMS ceramic and 9.7 ± 3.2 µm for the Curasan ceramic. The mean pore diameter of the RMS ceramics was determined to be 4.8 ± 1.2 µm and for the Curasan ceramics 10.9 ± 3.7 µm. In Figure 1a,b both ceramics are shown in a horizontal field width (HFW) of 46.6 µm. 

After incubation for 60 days in TRIS buffer (pH 5.0 or pH 7.4), the measurements were repeated. Strand width and pore sizes for the RMS and the Curasan ceramics are shown in Table 4. Furthermore, the influence of the TRIS buffer on the Curasan ceramics can be seen in Figure 1e,f. The altered surfaces are clearly visible in contrast to the untreated specimens. At pH 5.0, the crack propagation within the ceramic can be clearly seen, which ultimately led to the disintegration of the ceramic.

#### 3.2.2. Elementary Analysis by Means of EDX

**Figure 2:**
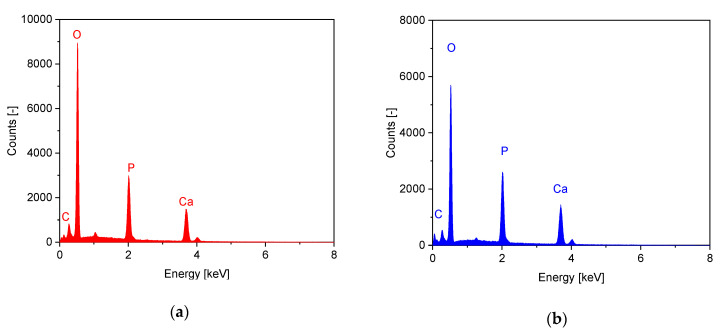

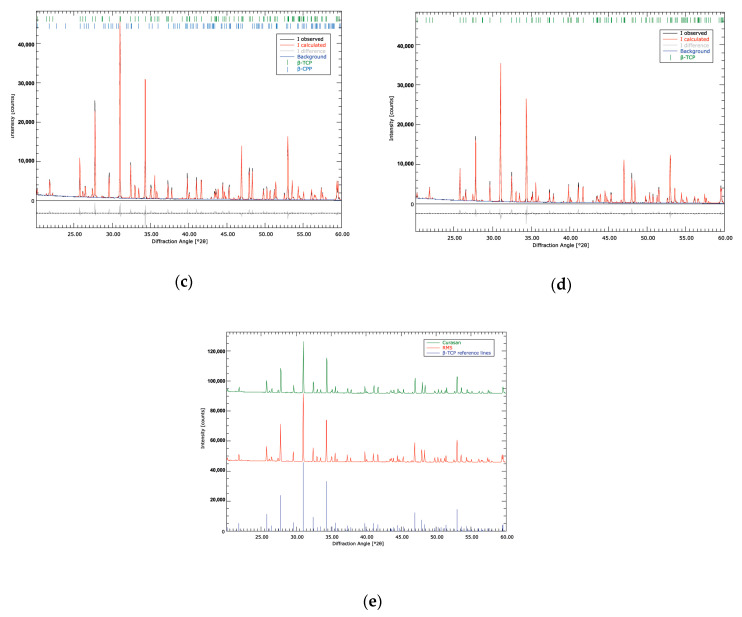
Dispersive X-ray spectroscopy (EDX) spectra of (**a**): RMS ceramics; (**b**): Curasan Ceramics; XRD pattern of (**c**): RMS ceramics; (**d**): Curasan ceramics; (**e**): XRD pattern RMS, Curasan with reference lines for β-TCP; EDX measurements carried out at Philips XL 30 FEG ESEM with EDX unit, 12 kV acceleration voltage and 300 s lifetime counting period; XRD measurements with Bruker D8 Advance, (measurement conditions: 40 kV/40 mA, 1°2 theta/min, step size 0.02°2 theta).

In the EDX measurements, traces of magnesium were found in the samples from the Curasan ceramics. The Ca/P ratio for the RMS ceramic was 1.49 and for the CUR ceramic 1.51. Thus, both samples appeared to be β-TCP (see Figure 2).

#### 3.2.3. X-Ray Diffraction Analysis

The XRD analysis with subsequent Rietvelt refinement analysis confirmed that both the Curasan and RMS ceramics consisted of β-TCP. In the RMS ceramic, traces of calcium pyrophosphate (from the manufacturing process < 1 wt %) were also identified (see Figure 2).

#### 3.2.4. Porosimetry

**Figure 3:**
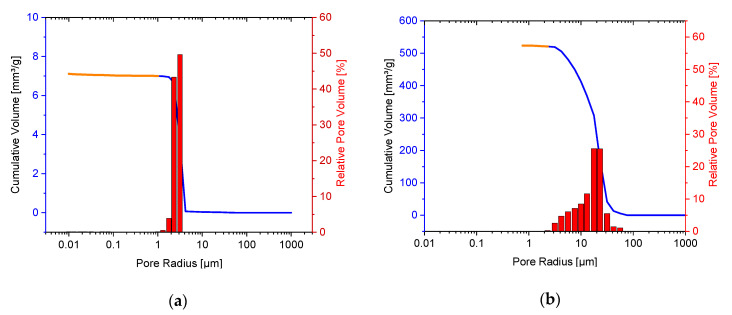
Pore size distribution (**a**): RMS ceramics and (**b**): Curasan ceramics.

The pore size distribution of the RMS β-TCP ceramics was determined with a mercury porosimeter Pascal 140 and 440. In the low-pressure range in Figure 3, the blue line was shown with the Pascal 140 under CO_2_ addition. The orange line in Figure 3 was obtained in the high-pressure range with the Pascal 440 under the addition of mercury. The two methods were used to show the presence of small pores of approximately 1 μm. The mean pore radius was 2.36 μm. The total porosity of the ceramic was 45.90%. In comparison, the mean pore radius of Curasan ceramics was 18.50 μm, and the total porosity 61.81%. In addition, Figure 3 shows that the variation in pore diameter was in the range of 3 to 80 μm, whereas the pores of the RMS were in a narrower range between 2 and 5 μm.

#### 3.2.5. Surface Roughness

**Figure 4:**
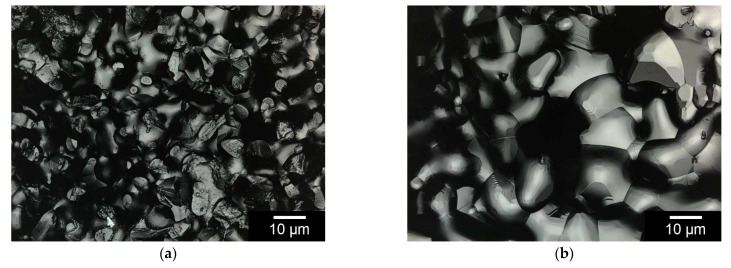

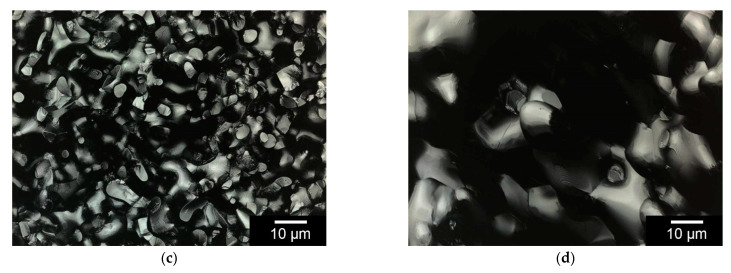
3D laser scanning microscopy images of the surface of (**a**): RMS and (**b**): Curasan Ceramics, Inner surface after incubation in TRIS buffer pH 7.4 for 60d (**c**): RMS and (**d**): Curasan ceramics; white bar represents 10 µm, Images taken with KEYENCE VK-X210.

Five different samples of each ceramic were examined. For each sample, the surface roughness parameter Sa was determined from four individual measurements on the surface. The measured values of the different samples are shown in Table 5. Using ANOVA, no significant difference in surface roughness could be detected between the different samples. Figure 4 shows an example of 3D laser scanning microscopy images of RMS and Curasan ceramics.

No significant difference in surface roughness was observed after incubation in SBF. In contrast, incubation in TRIS decreased the surface roughness of the RMS ceramics to 4.4% of the initial value at pH 7.4 and 6.6% at pH 5.0. No significant difference was found between the two pH values (see Table 5).

#### 3.2.6. MicroCT

**Figure 5:**
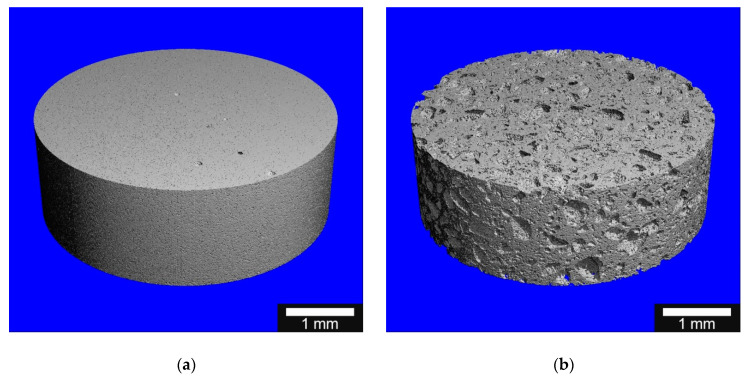

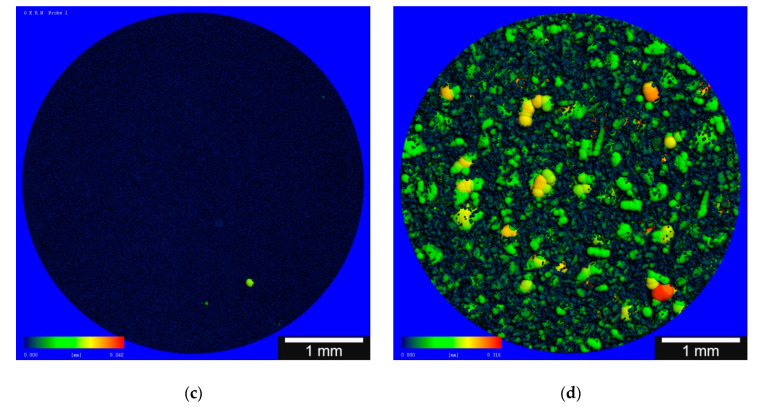
Micro-computed tomography (µCT) reconstruction of the different β-TCP ceramics: (**a**): RMS and (**b**): Curasan; µCT reconstruction of the porosity (**c**): RMS and (**d**): Curasan, the pores are displayed in false colors, the brighter, the larger the pores, the white bar corresponds to 1 mm, the colored bar goes from 0 (dark blue) to 0.316 mm (orange).

The differences in the porosity of the two different ceramics are already externally clearly visible (see Figure 5a,b). The reconstruction of the pores within the ceramic also showed clear differences. The RMS ceramic showed a uniform pore structure with occasional larger pores, whereas the Curasan ceramic had pores of different sizes from 0.001 to 0.316 mm (see Figure 5c,d). 

### 3.3. Degradation Experiments

#### 3.3.1. Degradation of β-TCP Ceramics

The degradation progress of β-TCP ceramics was measured by weight. The following Table 6 shows the start-, end-weights and the weight after loading with alginate or ADA-gelatin.

Table 6 shows the weight distribution of the ceramics at the start, after loading with alginate or ADA-gelatin, and at the end of the test. It can be seen that for all groups, the weight increased only by the loading weight of approximately 0.1 g. There were no further weight changes during the 60 days of testing. There was only a difference in weight between the two different ceramics. The Curasan ceramics had a significantly lower weight than the RMS ceramics. 

**Figure 6:**
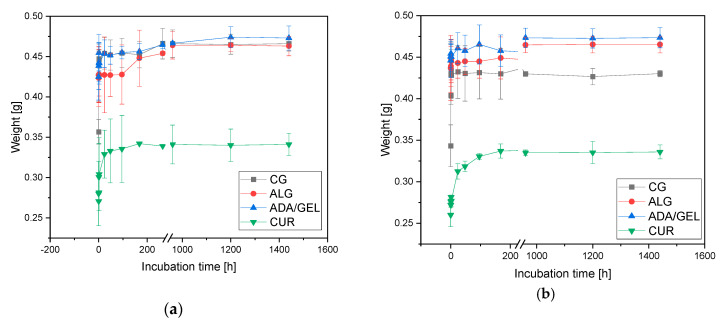
Weight development over the duration of the experiment; (**a**): incubation in TRIS pH 5.0, (**b**): incubation in TRIS pH 7.4; CG…control group = empty RMS; ALG…RMS + Alginate; ADA/gelatin … RMS + ADA/gelatin; CUR … empty Curasan.

Figure 6 shows the weight development of the samples at pH 5.0 and pH 7.4. The unfilled ceramics demonstrated a significant increase in weight within the first 4 h. Afterward, the weight remained constant. The control group (CG) and Curasan (CUR) samples showed an increase in weight due to liquid in the ceramics. After reaching full liquid loading, the weight did not change.

After the degradation experiments, the samples were again examined by 3D laser scanning microscopy. The results showed that for both the RMS and Curasan ceramics, the surface roughness and strand width decreased, and the pore size increased (see Table 4 and Figure 4). The degradation experiment in TRIS was intended to investigate the degradation behavior of ceramics. With our investigations, we were able to show that bulk degradation of the ceramics occurred, i.e., this degradation occurred in the entire ceramic. However, the degradation occurred in both ceramics because both ceramics were b-TCP. In both ceramics (RMS and Curasan), the strand width became smaller and the pores larger. In the case of the Curasan ceramic, this effect was intensified by the fact that the pores were significantly larger than in the RMS ceramic and, therefore, had a larger contact area with the TRIS solution. 

#### 3.3.2. Degradation ADA-Gelatin Hydrogel

**Figure 7:**
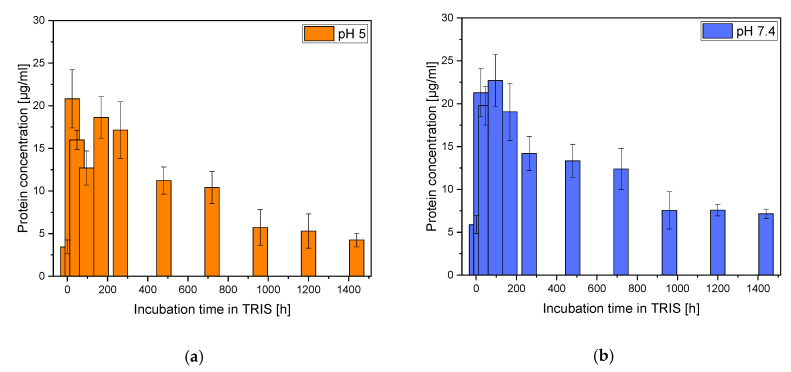
Overview of protein release out of the alginate–alginate-di-aldehyde (ADA-gelatin) hydrogel; (**a**): pH 5.0; (**b**) pH 7.4.

In terms of protein release, the samples incubated in TRIS pH 7.4 showed a slightly higher value than the samples incubated in pH 5 (Figure 7). Compared to the initial amount of proteins in the gelatin used, 54.6% were released by the 60-day incubation in TRIS pH 5.0 and 65.6% in pH 7.4.

### 3.4. Compression Test

**Figure 8:**
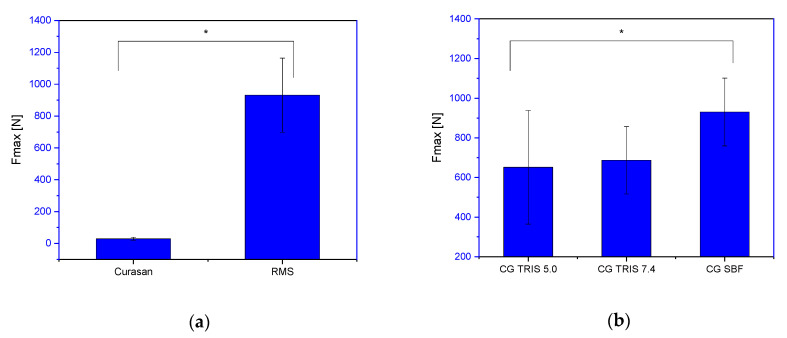

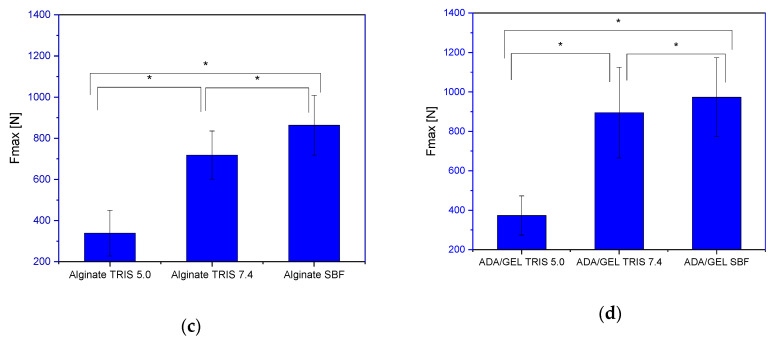
Maximum tolerated force for the different samples; (**a**): Comparison Curasan vs. RMS; (**b**): Control Group after incubation in different buffers for 30 (SBF) or 60 days; (**c**): Alginate loaded RMS ceramics after incubation in the different buffers for 30 (SBF) or 60 days; (**d**): ADA/GEL loaded RMS ceramics after incubation in the different buffers for 30 (SBF) or 60 days; the measurements was performed on ZWICK/Roell Z005 Universal Testing machine; * significant difference with *p* < 0.05.

The maximum failure load of the commercially available Curasan β-TCP ceramic was 29 ± 9.0 N, while the RMS ceramic was 931 ± 223 N. Comparing the values of the control group of the RMS ceramics, there were changes compared to the ceramics incubated 60 days in TRIS buffer with 686.55 ± 170 N. When incubated at pH 5.0, this change was even more pronounced, up to 651 ± 287 N. In contrast, the ceramics incubated in SBF solution again showed no significant change with 930 ± 171 N in the maximum failure load compared to the unloaded RMS ceramics of the control group (see Figure 8a,b).

The loaded ceramics showed a significant decrease in the maximum tolerated force during incubation for 60 days in TRIS pH 5.0. The RMS ceramics loaded with alginate tolerated only a maximum force of 339 ± 111 N, while the ADA-gelatin RMS loaded ceramics tolerated a 10% higher force, 373 ± 99 N. Incubation in TRIS pH 7.4 also demonstrated a decrease in the maximum force, but the difference to the initial value of the unloaded blanks was smaller at 931 ± 223 N. The alginate loaded RMS ceramics incubated in TRIS pH 7.4 showed a maximum failure load of 718 ± 117 N and the ADA-gelatin loaded RMS ceramics 895 ± 230 N (Figure 8c,d and Table 7). The maximum failure load for the alginate-loaded β-TCP ceramics and the maximum failure load of the ADA-loaded ceramics were significantly different with *p* < 0.05.

In addition, Table 7 shows that loading both alginate and ADA-gelatin led to an increase in the maximum failure load when incubated according to EN ISO 10993-14 standards in SBF pH 7.4. Compared to the unloaded ceramics, the maximum failure load decreased by 7.3% for ALG-loaded ceramics and increased by 19.7% for ADA-gelatin-loaded ceramics. 

## 4. Discussion

### 4.1. Dimensions

Both of the examined RMS and Curasan ceramics were made of β-TCP. However, they differed in their total porosity and pore sizes. The Curasan ceramic was macroporous with an average pore size of 37 µm and total porosity of 62%, whereas the RMS ceramic was microporous with an average pore size of 4.8 µm and total porosity of 46%. This explains the weight differences between the two ceramics. The determined surface roughness for both ceramics was not significantly different.

### 4.2. Elemental Analysis

The elemental analyses by means of EDX and XRD proved beyond doubt that the RMS ceramic, as well as the Curasan ceramic, were phase pure β-TCP. We have already performed similar verifications for RMS ceramics in the past [16,20,23].

### 4.3. Degradations Experiments

Contrary to expectations, the weight of the RMS ceramics did not change significantly after the end of the degradation experiments in TRIS pH 7.4 and TRIS pH 5.0. It remained at the level of the filled ceramics until the end of the experiments, with a weight difference of 0.1 g. However, the influence of the degradation experiment on the Curasan ceramics was clearly visible. In pH 5, the ceramics were dissolved after 60 days. In pH 7.4, the effect was not quite as strong. Nevertheless, one could see clear differences compared to RMS ceramics. Both ceramics (RMS, CUR) showed a decrease in surface roughness and strand thickness as well as an increase in pore size due to the effect of TRIS buffers. Similar to Boanini et al. [24], tendencies of the increase in weight of the loaded ceramics within the first 2 h were observed. However, these tendencies lasted only 2 h and were not significant. Liu et al. [25] performed similar degradation experiments in TRIS buffer pH 7.4, but only for 42 instead of 60 days as in our project. Their samples were also not filled with hydrogels. The β-TCP ceramics also only showed a weight loss of 1–2%, just like our samples. Shao et al. [26] also performed degradation experiments with TRIS buffer pH 7.4 over 42 days for their 3D constructions made of magnesium-doped wollastonite/-TCP bioceramic. Pure β-TCP ceramics were used as a control for their experiments. After 6 weeks, they showed a weight loss of 2%, which is similar to our data. However, as with Liu et al. [25], the constructs were not loaded with hydrogels. Ni et al. [27] observed only etched surfaces on β-TCP ceramics in their 28-day degradation experiments using calcium silicate, dimagnesium silicate, or tricalcium phosphate bioceramics in TRIS buffer with pH 7.4. They also reported a weight loss of approximately 2%. Faruq et al. [28] reported on degradation experiments with CaP granules loaded with hyaluronic acid/gelatin hydrogels but using a different degradation approach with lysozyme rather than the TRIS buffer. The experiment ran exactly like ours, for 60 days. However, only the dry weights of the hydrogels were determined afterward degradation. Nguyen et al. [29] reported on degradation experiments of hydrogen microspheres. However, these microspheres were only incubated in PBS, and dry weights were determined at defined time points. In addition, the pH changes of the medium were recorded. Distler et al. [30] determined the degradation behavior of ADA-gelatin plates. For this purpose, the gels were incubated in Dulbecco’s modified Eagle medium with 10% *v*/*v* fetal calf serum and 1% *v*/*v* PenStrep. Every 48 h, the medium was changed and analyzed. Gelatin release was analyzed by the Lowry method [31]. Unfortunately, Distler et al. [30] only studied the release of gelatin over 16 h. The courses of protein release showed a larger value for pH 7 than for pH 5. Kang et al. [32] reported similar observations in their experiments with chitosan–gelatin microparticles.

### 4.4. Compression Test

The results of the mechanical tests showed a difference between the β-TCP ceramic with ALG vs. with ADA/GEL. However, there were no significant differences between the empty RMS ceramics and the ceramics filled with ALG. There was only a significant difference between the empty RMS ceramics and those filled with ADA. Liu et al. [25] performed compression experiments on β-TCP ceramics. However, these constructs were incubated in TRIS for 42 days only and mechanically characterized after 6, 12, and 18 weeks. In contrast to our results, the 3D printed macroporous constructs of Liu et al. [25] showed a compressive strength of 11 MPa, whereas our microporous ceramics were characterized at 24 ± 6 MPa. After 18 weeks, the compressive strength decreased to 8.3 ± 1.2 MPa. In our experiment, the compressive strength decreased to 17.8 ± 4.4 MPa after 60 days in TRIS pH 7.4. In TRIS pH 5.0, the compressive strength decreased to 16.9 ± 7.5 MPa. Similar to the macroporous ceramic of Liu et al. [25], this results in a reduction to ~ 75% of the initial value. Torres et al. [33] coated a β-TCP/Hydroxyapatite ceramic with alginate. An increase in the compressive strength could be observed. Unfortunately, no values were given but only a reference to an image. In addition, in our project, the increase in the compressive strength could be observed by loading alginate or ADA-gelatin and was ~ 5% for alginate and ~ 8% for ADA-gelatin. Torres et al. [33] were of the opinion that the increase in the compressive strength is due to an increase in the wall thickness. We assume that the material properties of the ALG or ADA-gelatin also play an important role, especially the damping properties, in addition to the increase in the surface area by filling of the pores. Zhou et al. [34] came to the same conclusion. They investigated whether the shock absorption of intervertebral discs could be restored to a normal level by injecting hydrogels. It was shown that the intervertebral discs filled with hydrogel had better shock absorption than intervertebral discs without nucleus pulposus.

## 5. Conclusions

The composites made of ADA/gelatin gel loaded β-TCP ceramics have significant advantages (besides better degradability compared to alginate) over unloaded ceramics or alginate loaded ceramics, especially since the maximum failure load increased by ~20%. In addition, the TRIS buffer used in accordance with ISO EN 10993-14 indicated that the composites were quite strong. Nevertheless, it was shown that, especially at pH 5, there was a massive decrease in the maximum tolerated loads.

## Figures and Tables

**Table 1:** Composition of TRIS-buffered simulated body fluid (SBF) 27.

Reagent	Quantity [g]
Sodium chloride	6.547
Sodium hydrogen carbonate	2.268
Potassium chloride	0.373
Di-sodium hydrogen phosphate dihydrate	0.178
Magnesium chloride anhydrous	0.142
Calcium chloride dihydrate	0.368
Sodium sulfate	0.071
TRIS	6.057
1 M HCl	Until a pH 7.4 was reached

**Table 2:** Sample distribution of the β-TCP ceramics for the compression test.

	SBF	TRIS pH 7.4	TRIS pH 5.0
ADA-gelatin	15	30	30
Alginate	15	30	30
Control group	15	10	10
Curasan	15	15	15

**Table 3:** Parameters for the compression tests.

Geometry of the Sample	Round Sample
Tool distance at start position	250 mm
Speed start position	50 mm/min
Forward force	1 N
Speed of the preload	50 mm/min
testing speed	1 mm/min
Upper force limit	5000 N
Maximum deformation	50%

**Table 4:** Strand width and pore diameter of the different ceramics.

**0 d**		
**Blanc**	**RMS**	**CUR**
Strand width (µm)	7.1 ± 2.2	9.7 ± 3.2
Pore diameter (µm)	4.8 ± 1.2	10.9 ± 3.7
**60 d**		
**pH 7.4**	**RMS**	**CUR**
Strand width (µm)	3.6 ± 0.2	8.8 ± 4.1
Pore diameter (µm)	6.8 ± 2.2	12.6 ± 2.3
**pH 5.0**	**RMS**	**CUR**
Strand width (µm)	3.3 ± 0.1	6.7 ± 0.1
Pore diameter (µm)	8.7 ± 1.7	16.1 ± 4.1

**Table 5:** Surface roughness of different samples.

**Sa [µm]**					
**Before incubating in SBF/TRIS**					
Sample	RMS *	CUR	RMS-SBF	RMS-TRIS 7.4	RMS-TRIS 5.0
Mean ± SD	3.26 ± 1.1	6.34 ± 3.36	3.32 ± 0.46	3.68 ± 0.65	3.50 ± 0.87
**After incubating in SBF/TRIS**					
		0.25 ± 0.14	3.05 ± 0.96	0.16 ± 0.04	0.23 ± 0.10

* the control group was not incubated in SBF or TRIS.

**Table 6:** Weight development of the different ceramics over the experimental period.

**Weight [g]**			
**Empty Ceramics**	SBF *	**TRIS pH 5.0**	**TRIS pH 7.4**
CG	0.364 ± 0.006	0.359 ± 0.008	0.360 ± 0.07
ALG	0.360 ± 0.007	0.362 ± 0.008	0.359 ± 0.008
ADA/GEL	0.362 ± 0.008	0.365 ± 0.01	0.360 ± 0.007
CUR	0.272 ± 0.005	0.280 ± 0.04	0.260 ± 0.014
**Weight after loading**	**SBF**	**TRIS pH 5.0**	**TRIS pH 7.4**
Alginate	0.464 ± 0.024	0.463 ± 0.012	0.459 ± 0.012
ADA/gelatin	0.469 ± 0.019	0.469 ± 0.023	0.457 ± 0.013
**End Weight**	**SBF**	**TRIS pH 5.0**	**TRIS pH 7.4**
CG	0.479 ± 0.027	0.466 ± 0.009	0.459 ± 0.009
ALG	0.463 ± 0.008	0.463 ± 0.012	0.465 ± 0.01
ADA/gelatin	0.469 ± 0.019	0.473 ± 0.013	0.473 ± 0.015
CUR **	0.260 ± 0.005	n.a. ***	0.224 ± 0.007

* the SBF experiment only ran for 30 days; ** the Curasan ceramics were unfortunately too fragile for loading, so they only served as a control; *** the Curasan ceramics were destroyed after immersing in TRIS pH 5, only a few small granules were left; CG…control group = empty RMS; ALG…RMS + Alginate; ADA/gelatin … RMS + ADA/gelatin; CUR … empty Curasan.

**Table 7:** Maximum Failure Load and Compressive Strength of the Ceramics.

**Maximum Failure Load [N]**			
**Sample**	**Control Group**	**RMS + Alginate**	**RMS + ADA/GEL**
TRIS pH 5.0	651 ± 287	339 ± 111	374 ± 99
TRIS pH 7.4	687 ± 170	718 ± 117	895 ± 230
SBF	930 ± 171	863 ± 145	973 ± 200
No buffer	931 ± 223	863 ± 82	1114 ± 300
**Compressive Strength [MPa]**			
**Sample**	**Control Group**	**RMS + Alginate**	**RMS + ADA/GEL**
TRIS pH 5.0	17 ± 7	9 ± 0.3	10 ± 3
TRIS pH 7.4	18 ± 4	19 ± 3	23 ± 6
SBF	24 ± 4	22 ± 4	25 ± 5
No buffer	24 ± 6	22 ± 2	29 ± 8

## Data Availability

The data presented in this study are available on request from the corresponding author.
